# Supplementation with Polyunsaturated Fatty Acids as the Main Dietary Factor Is Associated with the Omega-3 Index in Lithuanian Professional Athletes

**DOI:** 10.3390/nu17243840

**Published:** 2025-12-08

**Authors:** Marius Baranauskas, Ingrida Kupčiūnaitė, Jurgita Lieponienė, Rimantas Stukas

**Affiliations:** 1 Faculty of Biomedical Sciences, State Higher Education Institution Panevėžys College, 35200 Panevėžys, Lithuania; ingrida.kupciunaite@panko.lt (I.K.); jurgita.lieponiene@panko.lt (J.L.); 2 Department of Public Health, Institute of Health Sciences, Faculty of Medicine, Vilnius University, 01513 Vilnius, Lithuania; rimantas.stukas@mf.vu.lt

**Keywords:** athletes, dietary habits, nutritional intake, polyunsaturated fatty acids, public health

## Abstract

**Background/Objectives**: Nutrition is essential for both physiological and physical health. The study aimed to explore dietary habits, nutritional intake and supplementation in association with the indirect omega-3 index (ω-3I) magnitude in a cohort of professional athletes. **Methods**: A 3-day food record was used as an approach to document all the dishes and beverages consumed by athletes over three consecutive days. Additionally, in aiming to assess the dietary habits and supplementation as well as the estimated ω-3I, both a food frequency questionnaire and a valid equation proposed by Swiss scientists were applied. The body composition of athletes was assessed using the bioelectrical impedance analysis. **Results**: Given that carbohydrate-containing foods were relatively frequently consumed by athletes, the average daily carbohydrate content (5.5 g/kg of body weight/day) did not reach the minimum recommended limit. A Western pattern diet applied to professional athletes ensured a sufficient level of protein intake (1.7 g/kg of body weight/day) and resulted in the overconsumption of dietary fat (40.3% of energy intake (EI)), especially saturated fatty acids (FAs) (13.8% of EI). The frequency of the consumption of fish products was considered to fulfill the lowest rank, which in turn, led to the lowest average intakes for polyunsaturated (6.2% of EI), ω-6 (5.7% of EI), and ω-3 (0.3% of EI) FA. Also, the dietary ω-3 FA deficiency generated an unhealthy ω-6/ω-3 FA ratio of 18.4:1 which was not directly related to the intermediate-desirable level (5.5–9.8%) of the estimated ω-3I in a sample of professional athletes. **Conclusions**: Taking into account the predicted regression model which was adjusted for sports and sex, the estimated ω-3I was significantly and positively associated with the higher consumption of polyunsaturated FA supplementation (β 1.5, 95% confidence interval (CI): 1.3; 1.6, *p* < 0.001), fish products (β 1.1, 95% confidence interval (CI): 1.0; 1.2, *p* < 0.001), and the energy percentage obtained from the dietary ω-3 FA (β 0.8, 95% CI: 0.1; 1.6, *p* = 0.049) in a cohort of professional athletes. Therefore, whilst acknowledging that the increased fish consumption may serve as an equally strong potential predictor for the indirect ω-3I magnitude, the supplementation with polyunsaturated FA also becomes an important strategy for maintaining the optimum ω-3I levels among professional athletes.

## 1. Introduction

Nutrition is essential for both physiological and physical health. Maintaining a proper nutrition throughout life promotes one’s healthy lifestyle and contributes to better development. To ensure bodily needs, it is essential to practice a balanced diet containing the necessary amounts of all macronutrients and trace elements [1]. For example, omega-3 (ω-3) fatty acids (FAs) play a potential role in preventing cardiovascular disorders and regulating the antioxidant signaling pathway as well as modulating inflammation [2]. However, across time, dietary preferences have always been linked to the diversity of cultures around the world. For example, when analyzing the consumption of fish products containing polyunsaturated FA, previous studies have shown that the consumption of fish in Southern European countries was significantly higher compared to other parts of Europe. More indicatively, in Portugal [3,4] and Italy [5,6], the estimated average daily consumption of fish products was 44 g and 44.6–46.6 g, respectively. Meanwhile, almost twice as many fish products were consumed by the residents of France (34.3 g/day), Finland (30.5 g/day) [7], Denmark (18 g/day) [6], Lithuania (21.2 g/day) [8], and the Czech Republic (11.7 g/day) [6]. Thus, whilst red meat products were consumed too often, at the same time, the intake of fish and seafood was too low among the residents of the Nordic and Baltic countries [9].

Furthermore, healthy food for adults should consist of fresh vegetables and fruits (at least 400 g/day), excluding potatoes and other starch roots, nuts, legumes, and whole grains (e.g., brown rice, unprocessed maize, and oats). Also, in order to maintain a healthy diet, the World Health Organization (WHO) has provided some practical suggestions based on both limiting the consumption of sugar-sweetened beverages and replacing saturated and trans FA with polyunsaturated FA [1]. In this context, it is particularly important to reduce the dietary intake of saturated FA found in fatty red meat, butter, coconut, and palm oil, while the consumption of polyunsaturated FA, known as ω-3 FA and ω-6 FA (found in fish such as salmon or sardines, olive oil, nuts, and avocado, for example), should considerably increase [9,10,11].

Out of all the essential ω-3 FA, our paper largely focuses on alpha (ἀ)-linoleic acid which can serve as a predecessor to longer-chain ω-3 FA, like docosahexaenoic acid (DHA) and eicosapentaenoic acid (EPA). Meanwhile, the main ω-6 FA was linoleic acid, which in turn, may contribute as a precursor for arachidonic acid (ARA) biosynthesis [12,13]. It should be mentioned that ἀ-linoleic acid and linoleic acid are ranked as essential FA because they can only be derived from diet sources and cannot be endogenously synthesized in the body. Additionally, although other polyunsaturated FA (ARA, DHA, and EPA) can be endogenously synthesized from ἀ-linoleic acid and linoleic acid, the conversion is available in very small quantities. For example, in humans, ἀ-linoleic acid can be converted to both DHA and EPA by <5% and 5–10%, respectively. Therefore, EPA, DHA, and ARA must be obtained from diet and dietary supplements to meet the basic physiological demands for maintaining the immune, neural, and cardiovascular functions too [12,13,14,15,16,17]. Overall, to ensure that the omega FA serves the anticipated wellness gains [2,12,16,18,19,20], the ratio between the ingestion of ω-6 FA and ω-3 FA should be balanced and fluctuate between 4:1 and 1:1.

Specifically, in the field of sports medicine, polyunsaturated FA are important cell components that can support the optimal environment for the function of membrane proteins. In a cell membrane, both the degree and the length of polyunsaturated FA cells help to ensure the cell signaling, membrane’s fluidity, and the overall functionality of cells [21]. Thus, the FA structure in cell membranes not only indicates the dietary lipid consumption but is also shaped by FA metabolism and many other potential triggers [22,23]. In this case, physical loads may cause metabolic changes in the utilization of FA [24] and lead to alterations in the ω-3 FA and ω-6 FA membrane composition [25,26]. Generally, recent research has proposed that supplementation with ω-3 FA may increase the activity of the immune system through the biochemical route of COX-2 enzyme [26,27,28,29], affect cell fluidity, alter protein synthesis and ensure cellular function, accelerate post-workout regeneration and adaptation to physical exercise [30,31,32]. Nevertheless, engaging in endurance (aerobic) training [33,34,35,36,37,38,39] or strength–power (anaerobic) training [30,40] may reduce the bioavailability of ω-3 FA and lead to diverse consequences too.

In evaluating the bioavailability of ω-3 FA in the body, it is possible to assess both the dietary intake of ω-3 FA and the concentration that can be measured in lymph, serum plasma, and blood cells. When evaluating a long-term bodily supply with ω-3 FA, owing to their stability, polyunsaturated FA indices are often quantified in the red blood cell (RBC) membranes and serve as the benchmark marker [41,42,43]. However, this method is not only expensive, requiring complex handling of blood samples, but also invasive. Therefore, for public health purposes, there is another indirect way to calculate the bioavailability of ω-3 FA in RBC membranes, which is expressed as the ω-3 index (ω-3I) [44]. The ω-3I is the proportion (in terms of the percentage) of the sum of DHA and the EPA content in the total FA composition in the RBC membranes and, based on the long lifetime and the abundance of RBC, acts as a good indicator of the ω-3 FA bioavailability for the past 80–120 days [12].

**Figure 1:**
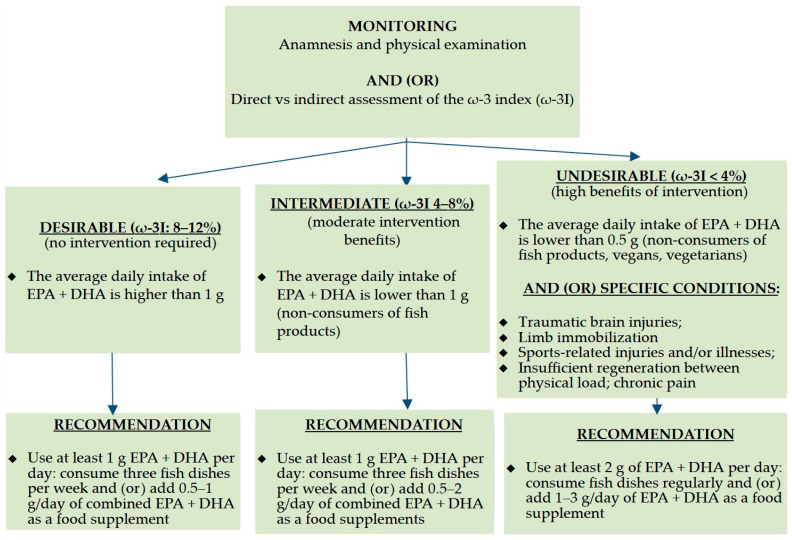
The recommended algorithm for the consumption of ω-3 fatty acids depending on the ω-3I (%) [44,46,47,48,49,50,51,52]. DHA—docosahexaenoic acid, EPA—eicosapentaenoic acid, ω-3I—ω-3 index.

It should be highlighted that ω-3I was initially suggested as an indicator to assess the mortality risk from coronary heart disease [45]. For this protective instance, the recommended level for ω-3I was roughly 8%, while the levels below 4% were related to an elevated risk of illness; however, these cut-offs have not yet been established based on in-depth research [46]. On the other hand, as presented in Figure 1, the recommendations for athletes accessible in the scientific field were constructed depending on the ω-3I.

The occurrence of training-related changes in the concentration of polyunsaturated FA, triggered by various structural and regulatory functions, may affect the athletic performance and health of both elite and non-professional athletes, stressing the importance of a proper intake of polyunsaturated FA with food along with an adequate supplementation as an additional strategy to ensure the desirable levels of ω-3I [53,54,55]. Furthermore, from a public health point of view, given that the bioavailability of ω-3 FA relies upon athletes’ dietary habits, nutritional profiles and the use of dietary supplements, the assessment of a complex relationship between the nutritional elements mentioned above and ω-3I estimated via a non-invasive approach [44] becomes particularly significant in a timely fashion. In this connection, emphasis must be laid on a high sensitivity of the cohort of professional Lithuanian athletes who, due to daily physical workloads, constantly strive for the highest results in both national and international sporting competitions. Thus, in order to optimize the sports nutrition in the Baltic States, the sample of professional Lithuanian athletes was particularly important in the design of this epidemiological study. Finally, the study aimed to explore the dietary habits, nutritional intake and supplementation in association with the indirect omega-3 index magnitude in a cohort of professional athletes. The following research hypotheses (in terms of the alternative hypothesis (H_a_) and the null hypothesis (H_0_)) were designed.

**H**_*a*_: *Dietary habits, nutritional intake and supplementation can assume the role as the independent predictive factors for the indirect ω-3I index magnitude in a cohort of professional athletes.*

**H**_*0*_: *Dietary habits, nutritional intake and supplementation have no association with the estimated ω-3I index magnitude in a sample of high-performance athletes.*

## 2. Materials and Methods

### 2.1. Study Design, Participants and Data Collection

A cross-sectional study conducted from March through September 2021–2022 (a macrocycle preparatory phase) involved a total of 322 professional Lithuanian athletes of qualified population pooled from the Olympic roster for Lithuania administrated by the National Olympic Committee (LTOK). An a priori representative sample size (n = 176–210) with a 95% confidence level, along with a margin of error of ±4–5% was set from the sample of well-trained athletes using the web-based and open-source program OpenEpi version 3.01 [56].

**Figure 2:**
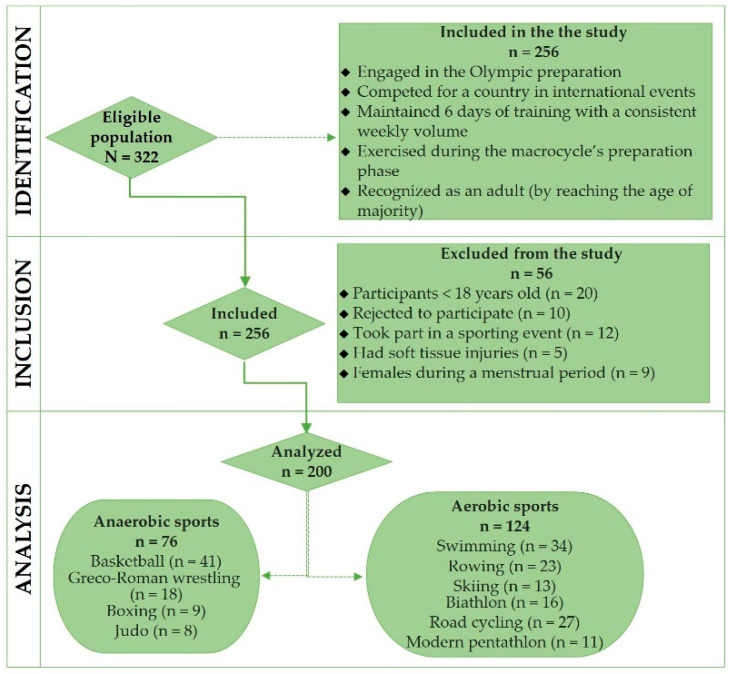
Enrollment process flowchart.

A simple random sampling technique was applied to recruit athletes for the cross-sectional study (Figure 2).

The main benchmarks for the admission of athletes in the study matched the following features: (1) participants who were recognized as an adult (by reaching the age of majority); (2) well-trained athletes who engaged in the Olympic preparation; (3) participants who competed for their country in national or international events; (4) athletes who maintained 6 days of training with a consistent weekly volume and exercised during the macrocycle preparatory phase. The criteria for ineligibility were established as follows: (1) participants who rejected the involvement in the observational study (n = 10); (2) athletes who took part in a sporting event (n = 12); (3) study participants who experienced soft tissue injuries (n = 5); (4) females during a menstrual period (n = 9); (5) athletes who have not reached the age of majority (n = 20). A more detailed procedure for the enrolment process is displayed in Figure 2.

Eventually, during the period from 2021 to 2022, 200 professional anaerobic (n = 76) and aerobic (n = 124) athletes who practice Olympic disciplines, namely, basketball, Greco-Roman wrestling, judo, boxing, swimming, rowing, skiing, biathlon, road cycling, and modern pentathlon were contained in the cross-sectional study (Figure 2).

### 2.2. Characteristics of the Athletes

In cooperation with a sports nutrition professional, the sports-related characteristics of athletes have been examined using the direct interviews at the Lithuanian Sports Centre. The questionnaire comprised the items related to the demographics and the aspects of athletic performance, namely, cultivated sport, sex (male vs. female), age (in years), training experience (in years), exercise per week (in days), single exercise sessions per day, duration of training (min per day), etc. Table 1 shows more detailed sports-related characteristics for professional athletes.

The mean age was 19.9 ± 2.3 years and the majority of study participants were males (n = 153). In the sample under analysis, the athletes were engaged in both aerobic (38%) and anaerobic (62%) sports. The elite careers of athletes lasted close to 7.9 ± 3.8 years. The average number of sports training sessions per week, daily workouts, and the time spent on physical exertion per day was 5.9 ± 0.8, 1.6 ± 0.5, and 180 ± 62 min, respectively. Taking into account the zones of exercise intensity, the levels of physical activity of elite athletes were fully in line with the training plans designed and approved by both the LTOK and Lithuanian Sports Centre (Table 1). More specifically, anaerobic and aerobic athletes devoted 38–60 and 55–78% of the total training time to physical loads in the strength endurance intensity zone. Meanwhile, in terms of the development of cross aerobic–anaerobic glycolytic strength capacity, the athletes’ time ratio of strength–power and endurance accounted for 32–49 and 15–35%, respectively. Furthermore, in both the anaerobic glycolytic strength and the adenosine triphosphate–phosphocreatine intensity zones, the workload of athletes matched 1–10% of the total amount of the whole training set.

### 2.3. Assessment of Dietary Habits and Use of Nutritional Supplements

The data on the athletes’ dietary intake, habits, and supplementation were collected through conducting a periodic medical examination and direct interviews with study participants, documented via athletes’ consultations with the assistance of a sports dietitian at the Lithuanian Sports Centre.

For the assessment of dietary habits and supplementation, a validated food frequency questionnaire established by Baranauskas [57] was applied. The questionnaire consisted of two main domains, which enabled the identification of: (1) the frequency of food consumption, classified into 14 groups, namely, breads, fresh vegetables and fruits, cooked potatoes, cereal products, dairy, confectionery, drinks with added sugar, poultry, egg, pork, beef, fish products, and semi-processed meat products (in terms of days a week); and (2) the duration of the consumption of 9 individual dietary supplements, namely, carbohydrates, amino acids, vitamins, minerals, PUFA, creatine, carnitine, caffeine, and herbal supplements (in terms of months a year). A four-point Likert-type scale with four response options (1—‘non-consumer of food product’, 2—‘1–2 days a week’, 3—‘3–5 days a week’, and 4—‘6–7 days a week’) was used to assess the frequency of food consumption. Also, a six-point Likert-type scale was used to measure the athlete’s frequency of dietary supplementation with six ordered response options as follows: 1—‘non-user of food supplement’, 2—‘less than 1 month a year’, 3—‘2–3 months a year’, 4—‘4–5 months a year’, 5—‘6–8 months a year’, and 6–‘more than 8 months a year’. For scales related to dietary patterns and food supplementation, the Cronbach’s ἀ reliability estimates were 0.71 and 0.79, respectively.

To interpret the average reported values of the food frequency consumption scale, the following sequence was applied: ‘0–2 days a week’ (1–2 points), ‘3–5 days a week’ (≥2–3 points), and ‘6–7 days a week’ (≥3–4 points). To elucidate the average declared values of the consumption duration for individual dietary supplements, the following rankings were used: 0–1 months a year (1–2 points), 2–5 months a year (≥2–4 points), and 6–12 months a year (≥4–5 points).

### 2.4. Dietary Intake Assessment

The 3-day food record as an approach was used to document all the dishes and beverages consumed by athletes over three consecutive days, seeking to evaluate a habitual pattern of food consumption and the levels of nutritional intake [58]. The special Atlas of Foodstuffs and Dishes [59], as a necessary tool for study purposes, was applied to record the specific food items consumed by well-trained athletes. During the investigation process, additional information related to consumed food portions (utilizing weight or measures), meal preparation methods, and food brands, including mealtime strategy, was also recorded. Furthermore, for the identification of the amounts of the main macronutrients consumed by athletes, this study used the Nutrition Baseline software (NutriSurvey, the English translation of a commercial German software (EBISpro version 3.02), SEAMEO-TROPMED RCCN-University of Indonesia) [60] with a manually integrated dataset of the foodstuffs’ nutritional values, which were originally from the Lithuanian food chemical composition data tables [61]. Consequently, the energy intake (EI) and the average amounts of dietary macronutrient fractions, namely, carbohydrates, protein, fat, and, including the ω-6/ω-3 FA ratio, consumed saturated, polyunsaturated, ω-3, and ω-6 FA by athletes were estimated.

The intakes of nutrients by the study participants were assessed in accordance with the recommended dietary allowances proposed by the research-based suggestions [62,63,64,65,66]. Hence, for athletic populations, the sufficient protein intake is equivalent to 1.2–2.0 g/kg of body weight (BW), the carbohydrate intake must correspond to 7–10 g/kg of BW, and the dietary fat percentage of the total daily calories should range from 25 to 35%. In terms of the recommended dietary allowances for the percentages of specific dietary FA fractions, namely, saturated, polyunsaturated, ω-6, and ω-3 FA, they must correspond to <10, 6–10, 5–8, and ≥2% of EI, respectively. Additionally, the ω-6/ω-3 FA ratio should fulfil a healthy interval, which held to be between 4:1 and 1:1 [67]. In order to compare the EI (kcal/day), this study also calculated the daily energy expenditure (kcal/day) in all athletes with reference to the recommendations established by Harris and Benedict [68], Ainsworth et al. [69], the American College of Sports Medicine and the American Dietetic Association, Dietitians of Canada [70] and the FAO/WHO/UNU [71].

### 2.5. Indirect Omega-3 Index Assessment

It should be highlighted that ω-3I is not influenced by acute dietary polyunsaturated FA intake or serious adverse clinical events; therefore, nowadays, ω-3I has been considered as a ‘low-noise’ parameter appropriate for use in epidemiological research [41,72,73]. In this connection, our study estimated ω-3I (%) by applying the valid equation proposed by Herter-Aeberli et al. [44], as follows:

Red blood cells (RBCs) total ω-3 (%) = 7.158 + (0.246 × dietary ω-3 FA intake) × (0.323 × sex) + (0.021 × age) + (1.612 × polyunsaturated FA supplement intake) − (1.874 × fish intake)

The estimation of the RBC ω-3 polyunsaturated FA composition was based on the dietary ω-3 FA intake (g/day). The age of athletes was measured in years, while sex was codified as female = 0 and male = 1. Supplement intake was evaluated by the following options: 0 = no polyunsaturated FA supplement, 1 = polyunsaturated FA supplement intake. Fish consumption as a variable was included in the equation as follows: 0 = fish consumption, 1 = no fish consumption.

Even though ω-3I was initially suggested as a biomarker to identify a life-threatening cardiovascular risk [45] and the appropriate level of protection for ω-3I was approximately 8%, while the levels below 4% were related to a higher exposition of adverse health-related outcomes, these thresholds have not yet been verified via experimental studies in design [45]. However, given that the specific standards on the ω-3I cut-off points have not been established, the recommended target variation for athletes as a proxy was considered to be 8–12% [47,48,49,50,51,52,74]. More specifically, our study categorized the levels of ω-3I risk thresholds into three subcategories as follows: (1) undesirable (ω-3I < 4%); (2) intermediate (4 ≤ ω-3I < 8%); and (3) desirable (8% ≤ ω-3I ≤ 12%).

### 2.6. Anthropometric Measures

The standing height (achieving ± 1 cm accuracy [75]) and BW (in kg) of athletes were assessed using the bioelectrical impedance analysis (BIA) [76] via the body composition analyzer X-Scan Plus (the International Organization for Standardization adopted by the European Union (EN-ISO): 13488 [77], Seoul, Republic of Korea) at the LSC. More explicitly, whilst the BIA approach was related to a four-compartment model in normal-weight adults [78], the analysis of the body composition of professional athletes was performed depending on the algorithms proposed by Lohman [75] and Kyle et al. [79]. Thus, when the X-Scan Plus device was operated at multi-frequency at 5, 50, 250, 550, and 1000 kHz, along with eight touch electrodes (in terms of two in each foot and two in each hand), the outcomes obtained from BIA were suitable to capture the core body components, such as body fat (in kg and % of BW) and fat-free mass (in kg and % of BW) too. The preciseness of weight measurements was guaranteed under the supervision of a sports dietitian throughout all the bioimpedance testing procedures. However, the auto-calibration of the X-Scan Plus tool was conducted each time when the weight measurements were imprecise. Additionally, athletes had to avoid sports practice (>24 h) before the evaluation process, as well as being requested to adhere to preliminary instructions: drinking no alcohol for 48 h and no liquids for 2 h, and emptying the bowels at least 30 min before testing.

The scales established by Skernevičius et al. were applied for the rating purposes of both fat-free mass and bodily fat percentage in athletes [80]. It has been thoroughly documented that sex-related physiological variations can contribute to disparities in body composition (in terms of size) among female and male athletes [81,82]. Thus, according to our study, the core components of both female and male athlete bodies, namely, fat-free mass and body fat percentage, were compared to the optimal reference values of 70–80, 75–85% and 20–24, 10–14%, respectively.

### 2.7. Statistical Data Analysis

For the normality of statistical data processing, the Shapiro–Wilk test was applied. The measures of central tendency (means (Ms) alongside 95% confidence intervals (CIs) and standard deviations (SDs)) were used to expose the gross values of the data under exploration. Secondly, for the bivariate analysis, with the intention to indicate the standardized difference between the two means, the *t*-test coupled with the calculated Cohen’s D (*d*) effect sizes were applied. The *d* values were elucidated as follows: 0.2 ≤ *d* < 0.5 (‘small effect’), 0.5 ≤ *d* < 0.8 (‘moderate effect’), and 0.8 ≤ *d* (‘large effect’). Furthermore, the multiple linear regression model was constructed to assess the association between the indirect ω-3I magnitude as a dependent variable and independent variables, namely, the nutritional intake, dietary habits and supplementation in a sample of professional athletes. For the linear regression model, the confounding variables as the covariates were set as follows: the sports branches and the sex of athletes. Additionally, the F-statistic and coefficient of determination (*R*-Squared (*R*^2^)) were evaluated to test the goodness-of-fit of the linear regression model. The critical value of the significance level was fixed as alpha (α) = 0.05 in all the statistical tests executed. The statistical data analysis was conducted by using the Statistical Package for the Social Sciences (IBM SPSS Statistics) version 25.0 for Windows (IBM Corp, Armonk, NY, USA). Also, the visual representation of the statistical data was performed by applying both the IBM SPSS software and the free and open-source software LibreOffice version 7.6.4.

Finally, the observational study was carried out in agreement with STROBE (strengthening the reporting of observational studies in epidemiology) checklist [83].

## 3. Results

### 3.1. Body Composition of Athletes

Given that sex-based physiological variations tend to result in the disparities of physical characteristics, Table 2 shows the study outcomes highlighting the differences between the core components of body composition among male and female athletes engaged in different sports. Although fat-free mass in male athletes was within the limits of the normative cut-offs, a significantly higher level of lean body mass was developed in a subgroup of anaerobic athletes compared to endurance male athletes (83.1 ± 4.8 vs. 82.7 ± 3.4% of BW, *d* = 0.4). In addition, regardless of the sport being cultivated, the body fat percentage in females (22.6 ± 3.5% of BW) varied within the optimum limits (20–24% of BW). Meanwhile, in terms of the large effect size (*d* = 0.8), the male body fat content (17.1 ± 4.0% of BW) exceeded the optimum upper bound of 14% and referred to a slightly higher body fatness (approx. 2.8–3.3% of BW) that, in turn, is not recommended for high-performance athletes. Table 2 provides more detailed information on the sporting and anthropometric characteristics based on sport branches and sex of athletes.

### 3.2. Dietary Habits and Supplementation

**Figure 3:**
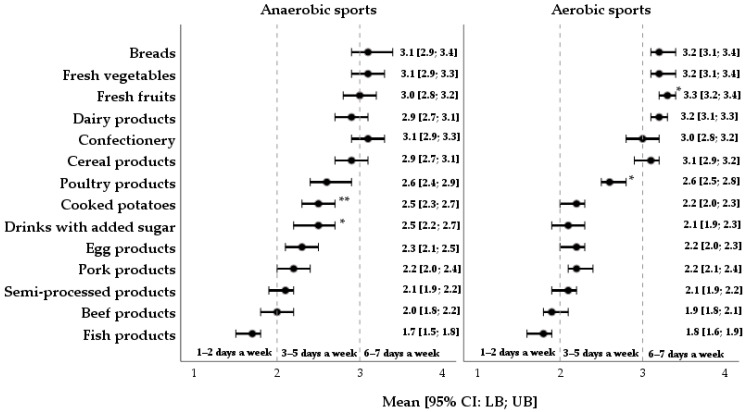
Eating habits and food choices in a sample of athletes competing in different sports. Data are presented as means ± 95% CIs [LB; UB]; *—*p*-value ≤ 0.05; **—*p*-value ≤ 0.01.

Figure 3 shows the results of the frequency of food consumption, which could undoubtedly be linked to the recommendations for a healthy diet. Regardless of sport, the highest consumption frequency of at least 6–7 days a week was indicated by athletes for breads, fresh vegetables and fruits, dairy products, confectionery, and cereal products. Meanwhile, athletes tended to consume unhealthy food items such as drinks with added sugar and semi-processed meat products less frequently, between 3 and 5 days a week.

It should be highlighted that fish products were consumed at the lowest frequency, i.e., only 1–2 times a week; however, as an alternative to food choices, study participants preferred to consume meat, namely, poultry, pork, beef, and egg products 3–5 times a week. Additionally, when comparing endurance athletes and anaerobic study participants, significant differences were found with regard to an increased consumption of fresh fruits (M_score_: 3.3 ± 0.8 vs. 3.3 ± 0.8; *d* = 0.4) and poultry products (M_score_: 3.2 ± 0.9 vs. 2.9 ± 0.9; *d* = 0.3). On the contrary, in terms of habitual consumption, strength–power athletes, compared to endurance athletes, more often tended to choose cooked potatoes (M_score_: 2.5 ± 0.9 vs. 2.2 ± 0.8; *d* = 0.4) and beverages with added sugar (M_score_: 2.5 ± 1.1 vs. 2.1 ± 1.0; *d* = 0.4).

**Figure 4:**
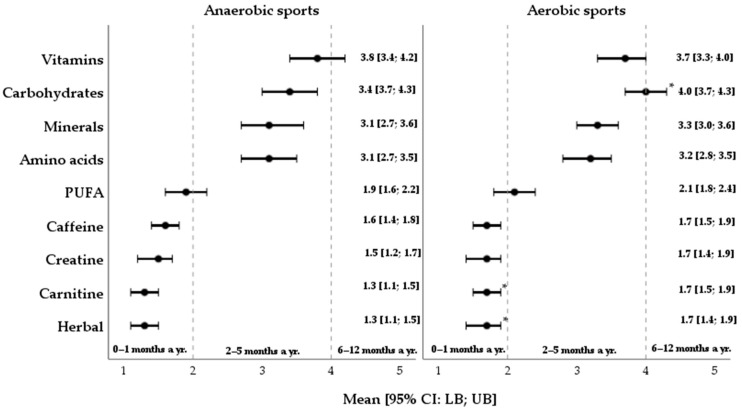
Dietary supplement consumption duration according to sports discipline. Data are presented as means ±95% CIs [LB; UB]; yr.—year; PUFA—polyunsaturated fatty acids; *—*p*-value ≤ 0.05.

The study assessed the duration of dietary supplementation use that served as one of the core components when evaluating the eating habits in athletes. As shown in Figure 4, vitamins, carbohydrates, minerals and amino acids were the most common dietary supplements used by athletes 2–5 months a year.

Meanwhile, the least frequent consumption of polyunsaturated FA supplements (M _score_: 2.0 ± 1.4) ranged from 0 to 1 month a year. Similarly, the athletes demonstrated a rare frequency of the consumption (<1 months a year) of caffeine, creatine, carnitine, and herbal supplements.

Additionally, a cohort of aerobic athletes revealed slightly higher levels of carbohydrate compared to the subgroup of anaerobic athletes (M_score_: 4.0 ± 1.7 vs. 3.4 ± 1.7; *d* = −0.4), carnitine (M_score_: 1.7 ± 1.2 vs. 1.3 ± 0.8; *d* = −0.4), and herbal supplementation (M_score_: 1.7 ± 1.3 vs. 1.3 ± 0.9; *d* = −0.3).

### 3.3. Nutritional Intake

Table 3 shows the reported usual mean intakes of energy, protein, carbohydrates, and FAs of the professional athletes. The average energy intake was 3551 ± 1191 kcal/day and ranged from 3338 to 3900 kcal/day in aerobic and anaerobic athletes. A carbohydrate-deficient diet (5.5 ± 1.9 g/kg of BW/day, consuming 45.3 ± 7.7% of daily calories) was usual in athletes.

Simultaneously, the average protein uptake of 1.7 ± 0.6 g/kg of BW/day fluctuated within the recommended boundaries of 1.4–2.0 g/kg of BW/day in a sample of professional athletes. Additionally, the study found that athletes compensate for carbohydrate deficits through the excessive consumption of dietary fat (40.3 ± 7.4% of EI) and saturated FA (13.8 ± 3.3% of EI). However, in food rations, the levels of polyunsaturated FA (6.2 ± 2.2% of EI) and ω-6 FA (5.7 ± 2.1% of EI) were barely below the minimum recommended limits. Finally, irrespective of the sport cultivated, athletes consumed too little ω-3 FA (0.3 ± 0.2% of EI). The dietary ω-3 FA deficiency, in turn, resulted in an unhealthy ω-6/ω-3 FA ratio that was equal to 18.4:1.

### 3.4. Indirect Omega-3 Index

**Figure 5:**
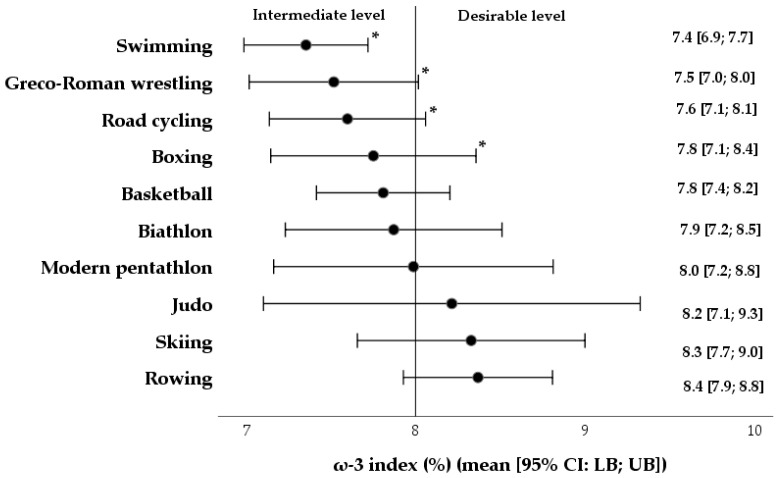
The central tendencies of the estimated ω-3 index (%) (in terms of risk thresholds), according to sports disciplines of athletes. *—*p*-value ≤ 0.05.

In terms of summing up the actual consumption of ω-3 FA and the frequency of fish consumption and the use of dietary supplements, the mean estimated ω-3I was 7.8 ± 1.2% and fluctuated between 5.5% to 9.8% in a sample of professional athletes. According to the estimated ω-3I outcomes, no significant differences were observed between aerobic and anaerobic athletes (7.8 ± 1.2 vs. 7.8 ± 1.2%; *d* = −0.03), including the subgroups of both male and female athletes (7.8 ± 1.1 vs. 7.9 ± 1.3%; *d* = −0.1). Nevertheless, as displayed in Figure 5, when compared to the lower bound of the norm (8–12%), the mean estimated ω-3I was in the desirable range among the athletes competing in sports, namely, rowing (8.4 ± 1.0%; *d* = 0.4), skiing (8.3 ± 1.1%; *d* = 0.3), judo (8.2 ± 1.3%; *d* = 0.1), modern pentathlon (7.8 ± 1.2%; *d* = −0.1), biathlon (7.9 ± 1.2%; *d* = −0.1), and basketball (7.8 ± 1.2%; *d* = −0.1). Concurrently, the cohorts of swimmers, Greco-Roman wrestlers, road cyclists, and boxers were within the moderate range, with the estimated ω-3I mean values of 7.4 ± 1.0, 7.5 ± 1.0, 7.6 ± 1.2, and 7.8 ± 0.8% (−0.6 ≤ *d* ≥ −0.3), respectively.

### 3.5. Omega-3 Index in Association with Nutritional Factors, Dietary Habits and Supplementation

In the final phase of the study data analysis, a multiple linear regression model was developed in order to predict the magnitude of the estimated ω-3I as a dependent variable variation (Table 4). Also, the linear regression model included independent variables, namely, the EI values (in %), which were supplied by the intake of both ω-3 and ω-6 FA, the ω-6/ω-3 ratio, the frequency of fish-derived products consumption, and the duration of polyunsaturated FA supplementation. Although the potential nutritional factors, dietary habits and supplementation may depend on the sports branches and sex of athletes, in the regression model, the characteristics mentioned above have been assigned as confounding variables.

The regression model indicated the 79% (*R*^2^ = 0.79, F_7_._192_ = 105.7) of the variance in the estimated ω-3I that can be explained by the independent variables, namely, the dietary ω-3 FA% of EI, the consumption of fish products, and the use of polyunsaturated FA supplements. More specifically, as shown in Table 4, the variations in the estimated ω-3I amid athletes were significantly and positively related to higher levels of the polyunsaturated FA supplements (β 1.5, 95% CI: 1.3; 1.6, *p* < 0.001), the higher consumption of fish products (β 1.1, 95% CI: 1.0; 1.2, *p* < 0.001) and the energy content (in %) derived from the dietary ω-3 FA intake (β 0.8, 95% CI: 0.1; 1.6, *p* = 0.049).

Additionally, the association between the estimated ω-3I and ω-6 FA intake (β 0.01, 95% CI: −0.1; 0.1, *p* = 0.768), along with the ω-6/ω-3 FA ratio (β −0.01, 95% CI: −0.03; 0.02, *p* = 0.576), was not revealed.

## 4. Discussion

### 4.1. Dietary Habits, Nutritional Intake and Supplementation

Given that cardiovascular and other dietary diseases are not only no longer increasing but also even decreasing in their prevalence in the Nordic countries such as Finland, Sweden, and Norway [9], the Dietary Guidelines for Lithuanians have indicated that the diet of the entire population must be changed by reducing the amount of saturated FA and sugar while promoting the consumption of vegetables, fishery products and whole-grain products [11]. However, based on the increased energy demand, especially from carbohydrates and proteins, the dietary habits and the use of macroelements among athletes differ from the general population as a whole in terms of diet quantity, time and specific goals (performance/recovery). The study found that athletes generally consumed breads, fresh vegetables, fruits, dairy products, confectionery, and cereal products for 6–7 days a week. To a lesser extent, between 3 and 5 days a week, athletes drank beverages with added sugar that are not considered to be a healthy food. Also, a more frequent consumption of drinks with added sugar and cooked potatoes was habitual among strength–power athletes. Meanwhile, the consumption of fresh fruits containing carbohydrates was more typical in a subsample of endurance athletes. In this context, in spite of a relatively frequent use of carbohydrate-containing foods by athletes, the average daily carbohydrate content (≈5.5 g/kg of BW/day) did not reach the minimum recommended limit (7 g/kg of BW/day). A similar situation referring to the insufficient consumption of cereal-based foods and carbohydrate deficits in the diets of athletes has also been identified in other countries, namely, Iran [84], Greece [85], France [86], Belgium [87], Poland [88,89,90] and Portugal [91]. Furthermore, the lack of carbohydrates is not recommended for athletes because the deficit may increase the fatigue of the central nervous system, adversely affect the immune system [92] and reduce aerobic capacity [93,94]. Considering the adverse consequences mentioned above, low-carbohydrate diets are consistently not recommended to professional athletes.

The study found that athletes quite often used high-protein and fat-rich foods such as dairy products (6–7 days a week) and poultry, pork, beef, and egg products (3–5 times a week). For professional athletes, this type of diet not only ensured a sufficient level of protein intake (≈1.7 g/kg of BW/day) but also resulted in the overconsumption of dietary fat (≈40.3% of EI), especially saturated FA (≈13.8% of EI). A consistent situation has also been identified for foreign professional athletes who have followed a high-protein diet and have adequately adhered to protein consumption requirements [91,95,96,97,98,99,100,101,102,103,104,105]. Nonetheless, the sample under analysis showed that low-carbohydrate diets were compensated by higher dietary fat intakes. It should be noted that fatty diets are not necessarily associated with adverse effects on the blood lipid profiles (e.g., plasma triglycerides and low- and high-density lipoprotein cholesterol) in the populations of athletes [106]. However, some research identified a link between the dietary saturated FA and the circulating homocysteine levels acting as a cardiometabolic risk factor [107,108]. Additionally, the potential benefits of a reduced intake of saturated FA may be justified by the outcomes obtained from other studies, which refer to the fact that a diet high in saturated fats is associated with an increased inflammation of the adipose tissue (in terms of via receptors such as TLR4) and muscle pain as well as inhibited regeneration [109]. Thus, our study deals with a significant issue associated with the nutritional objective to be attributed to athletes as a suggestion for a switch from a fat-enriched diet accounting for ~40% of total calories to a high-carbohydrate diet.

It is also important that the most typical dietary supplements used by athletes for 2 to 5 months a year are as follows: carbohydrates, amino acids, vitamins, and minerals. The scientific field has revealed that the use of dietary supplements (e.g., carbohydrate bars, gels, drinks and amino acids) may be recommended for professional athletes [70,110]. Therefore, according to our study, whilst most athletes did not consume enough dietary carbohydrates, compensating for these macronutrient deficits with dietary supplements can be considered a key strategy. On the other hand, the additional use of protein supplements could be more in line with the nutritional intake, physical workload plans (e.g., promotion of muscle hypertrophy) of athletes, a fortiori, the intake levels of dietary protein alone were sufficiently high. Thirdly, medicinal supplements (e.g., multivitamins, vitamins, minerals) should only be recommended for use in the case of deficiencies in these micronutrients and under medical supervision [70,110].

### 4.2. Omega-3 Index in Relation to Dietary Habits, Nutritional Intake and Supplementation

This study focused on the athletes’ dietary habits, supplementation and nutritional profile in relation to indirect ω-3I. Against this background, the frequency of the consumption of fish products observed in the cohort of the athletes under analysis stood at the lowest rank, i.e., between one and two times a week, which in turn, led to the lowest average intakes for polyunsaturated (≈6.2% of EI), ω-6 (≈5.7% of EI), and ω-3 (≈0.3% of EI) FA.

In the subsequent phases of the study, regardless of the sport being cultivated by professional athletes included in the sample, the estimated ω-3I was equal to the intermediate-desirable level (5.5–9.8%). The consistency was observed between the estimated ω-3I of the athletes we studied and that of the residents from other countries (Japan, South Korea, Alaska, and Spain; 6% < ω-3I > 8%) [111]. Meanwhile, the levels of ω-3I (4–6%) recorded in the residents’ of other countries, namely, the United States, Canada, Italy, and Germany were lower compared to professional Lithuanian athletes [111]. Similarly, when comparing the estimated ω-3I data derived from this study with the outcomes (the combined ω-3I ≈ 4.4%) obtained from research on 1452 athletes from many other countries, the difference referring to higher levels of indirect ω-3I among Lithuanian professional athletes was identified [112]. Although ω-3I is nowadays considered a ‘low noise’ marker advisable for the use in the epidemiological studies because it is not altered by an acute use of the ω-3I polyunsaturated FA or serious medical occurrences [113], the disparities mentioned above can be explained by the fact that our study has estimated ω-3I indirectly following the equation established by Herter-Aeberli et al. from Switzerland [44].

Secondly, multiple potential factors can have a small impact on ω-3I, including smoking [114,115,116], sex [115,117], age [114,115,118], genotype (accounts for ~24% of the ω-3I variation) [115,119], waist circumference [114,115], and body weight status [120], whereas nutritional status, especially the intake of sources of marine ω-3 FA foods and EPA + DHA-rich supplements, has a dominant effect on the ω-3I magnitude [114,115,116,120]. Moreover, there is scientific evidence that even short-term additional supplementation ranges (3–4 weeks) of higher concentrations of polyunsaturated FA have proven to be very effective in increasing ω-3I concentration [113]. Thus, according to our study, the use of polyunsaturated FA supplements by athletes for a shorter period of time (up to 4 weeks per year) may have had an impact on the maintenance of the estimated ω-3I for a suboptimal level. It should be highlighted that the benefits of the consumption of fish products and polyunsaturated FA for maintaining the estimated ω-3I were confirmed by the multiple linear regression model (adjusted for sport branches and sex) developed in this study. More characteristically, the variations in the indirect ω-3I among athletes were positively related to both the marine-rich food consumption and the polyunsaturated FA supplementation.

Furthermore, as regards the magnitude of ω-3I, sports where the estimated ω-3I level was below the desired bound must also be taken into account. In terms of the indirect ω-3I (7.4–7.8%), the samples of aerobic athletes (road cyclists, swimmers) and anaerobic athletes (wrestlers, boxers) were in the mid-range. Our study findings were consistent with the data published by Davinelli et al. [33] and Tepsic et al. [121] reporting the undesirable levels of ω-3I among runners and boxers. Also, dietary supplements containing ω-3I EPA and DHA have been shown to reduce the outcomes of inflammatory elements in athletes engaging in long-term and high-intensity exercising, namely, triathlon or marathon competitions [26,27]. In terms of the scientific field related to combat sports, the benefits of using ω-3 FA and its association with ω-3I have received little research. This study can only speculate that the malnutrition of wrestlers and boxers can be related to permanent (in terms of ≈ 7.1 episodes of weight reductions a year [122]) rapid body weight loss coupled with practiced weight loss techniques, such as restricting fluids and skipping one or two meals before the competitions in order to achieve the required weight category [123]. Therefore, the addition of polyunsaturated FA supplements to combat athletes’ food rations could be beneficial; however, further studies are necessary to confirm the inductive hypothesis.

### 4.3. Omega-3 Index and Omega-6/Omega-3 Fatty Acid Ratio

This study revealed that a dietary ω-3 FA deficiency generated an unhealthy ω-6/ω-3 FA ratio of 18.4:1 in professional athletes. The higher ω-6/ω-3 FA ratio was due to the higher consumption of ω-6 FA and the lower intake of ω-3 FA. Research on athletes in the United States [124], China [125], and Japan [126] has also established dietary habits in agreement with high levels of ω-6 FA intake. This imbalance can lead to significant physiological consequences because both FA families compete for the same metabolic enzymes and ω-6 FA may correlate with the elevated levels of pro-inflammatory markers (e.g., C-reactive protein and interleukin-6) [127]; therefore, the unbalanced ω-6/ω-3 FA ratio can serve as an additional trigger for exercise-induced cellular stress [128] and adversely affect several physiological systems, namely, muscular and cardiovascular systems, thereby modulating tissue regeneration and adaptation [33,47]. Thus, in this connection, some scientific studies have found that beyond underconsumption of ω-3 FA, an excessive ω-6 FA intake can exacerbate suboptimal ω-3I concentrations by disrupting the ω-6/ω-3 FA ratio [112]. However, being focused on comparison, this study did not identify the relationship between the estimated ω-3I and ω-6 FA intake or the ω-6/ω-3 FA ratio in a cohort of professional athletes. In this regard, although the higher intake of ω-6 FA may limit the inflammatory outcomes from ω-3 FA and cannot directly cause inflammation, the association between the ω-6/ω-3 FA ratio and their lipid intermediaries is mixed and has not been fully understood yet in a segment of athletes [129].

### 4.4. Strengths, Recommendations and Limitations

In terms of health-related recommendations, this study has for the first time highlighted the nutritional inequalities related to dietary habits and the ω-3 FA consumption in professional athletes from the Baltic States. In line with the general recommendations provided by the Nordic countries, given that red meat contains substances that increase the risk of cardiovascular disease and cancer, the dietary patterns of professional athletes should be modified and constructed by limiting the consumption of red meat (e.g., pork, beef, sheep meat, goat meat) to 350 g per week. As this study has detected the insufficient consumption of fish products by athletes, they are highly recommended to replace red meat and meat products with fish (i.e., a weekly intake of at least 300–450 g of fish can be proposed). As regards the consumption of marine-rich foods, the minimum recommended quantity should be at least 200 g per week of fatty sea fish (e.g., salmon, trout, mackerel or herring) that are the most enriched with ω-3 FA. Also, in this context, it is proposed to limit the consumption of predatory fish (e.g., redfish, hake, tuna, swordfish), as these fishes may contain mercury [11].

Additionally, taking into account the supplementation with polyunsaturated FA, especially ω-3 FA (EPA + DHA), in the cases where physical workloads are likely to affect the metabolism of FA [126], recent studies have identified only a modest [49] or no correlation [126] between dietary ω-3 FA intake and blood ω-3 FA levels among athletes [49] and proposed the hypothesis related to the standard dietary guidelines (250–500 mg EPA + DHA) which may not be sufficient for the target population. Hence, the International Olympic Committee (IOC) suggests using 2 g of ω-3 FA daily for professional athletes, in the cases where this dose of polyunsaturated FA supplementation significantly exceeds the general guidelines (250–500 mg/day) proposed for the entire population.

Finally, considering the fact that our study has identified the risk groups of road cyclists, swimmers, wrestlers, and boxers with the suboptimal estimated ω-3I levels, and also bearing in mind the scientific paradox that even athletes who comply with the proposed ω-3 FA dietary guidelines may have lower ω-3I levels than the general population [111], there is a vital need for professional athletes to receive a personalized ω-3 FA intake based on the routine ω-3I estimates coupled with dietary and educational interventions in a timely manner. In this framework, the training programs of athletes should be oriented to: (1) the routine testing for ω-3I every 80–120 days [12] and raising the awareness of athletes by delivering teaching provided by a sports dietician on the potential benefits of marine-rich foods and ω-3 FA; (2) the catering of organized and personalized food rations rich in fish (at least three times a week); (3) in the cases of an increased bodily demand, the provision of polyunsaturated FA supplementation (EPA + DHA).

There were several limitations in this study. Firstly, minor biases may have occurred due to the methodology used in the dietary evaluation process where the accuracy of the dataset may have been impacted by a range of confounders, namely, a memory deficiency in filling out dietary logs or a failure to identify food brands, diverse food beliefs or exact dosages of polyunsaturated FA (EPA + DHA) supplementation. Secondly, given that ω-3I may vary depending on both training intensity [113] and weekly volume [33], athletes might need higher ω-3 FA intakes during the periods of competition or intense training. Therefore, additional longitudinal studies at individual phases of the macrocycle could provide added value in filling the scientific gaps. Thirdly, for individual purposes, traditional dietary assessments along with indirect ω-3I estimates may not fully reflect the ω-3I status among athletes, highlighting the need for blood biomarker analysis for more precise evaluation [49,126].

Fourthly, taking into account both the high level of specificity of the target population due to the increased physical activity and a representative sample size of our study, all of this may, in turn, partly reduce the generalization of the results obtained from this study to a less physically active general population. On the other hand, it should be emphasized that the study opted to use the prediction equation developed and validated via ‘gas chromatography coupled to a tandem mass spectrometer (GC-MS/MS, Thermo Scientific TSQ 8000, Waltham, MA, USA)’ [44] for a general Swiss population as an indirect method to estimate ω-3I, instead of the direct, gold-standard measurement in RBC [111]. Nevertheless, the regression equation proposed by the Swiss scientists [44] did not contain a variable related to the physical activity component, which could lead to minor deviations in the estimated ω-3I measurements (in terms of general population vs. athletic population). In this case, with regard to the branches of athletic sports, our study highlighted significant differences that were controlled as the confounders during the phase of the data regression analysis. Therefore, for the sake of public health, with the intention to assess the ω-3I variations as well as taking into consideration that the direct application of the proposed equation to a specific cohort of Lithuanian elite athletes, who have distinct metabolic demands and training profiles, represents a significant extrapolation, further research vectors should be targeted to establish a validated regression equation exclusively in the populations of athletes.

Fifthly, the reference outcomes related to body composition in this study were estimated via BIA, which is considered to be ‘the indirect’ method for estimating body fat percentage. Given that study participants competing in some sports (e.g., combat athletes) can exhibit significant body fluid fluctuations during a training season, the BIA method may not be the most suitable for assessing body composition in athletes. Hence, depending on the potential non-uniform conductivity caused by factors such as hydration status or inter-individual differences in body composition, the gold standard may potentially necessitate other advanced techniques instead of the BIA method [130].

Finally, although the study was a cross-sectional in design, the results acquired from this study make no reflection of causal associations between the variables analyzed. Thus, further directions related to cohort and/or experimental studies in design could provide additional prominent scientific soundness.

## 5. Conclusions

Professional athletes should replace a typical Western pattern diet deficient of carbohydrates and omega-3 fatty acids but full of predominantly saturated fatty acids with a health-friendly diet that contributes to the achievement of sports nutrition goals. Moreover, taking into account the predicted regression model which was adjusted to sports and sex, the estimated omega-3 index was significantly and positively associated not only with the higher consumption of fish products but also with the supplementation by polyunsaturated fatty acids and the energy percentage obtained from the dietary omega-3 fatty acids in a cohort of professional athletes from the Baltic States. For this particular instance, athlete development programs should concentrate on testing the omega-3 index, professional training on the benefits of omega-3 fatty acids and, if possible, as a part of the catering arrangement, serving fish meals consisting of rich sources of eicosapentaenoic and docosahexaenoic acids alongside micronutrients and high-quality protein (e.g., salmon, herring, anchovies, and sardines) [131,132] at least three times a week.

Furthermore, although the lack of consumption of marine-derived omega-3 fatty acids is a potential trigger leading to suboptimal omega-3 index levels, the dietary intake alone may not ensure the optimal bodily demands for omega-3 fatty acids. Therefore, whilst acknowledging that the increased fish consumption may serve as an equally strong potential predictor for the indirect omega-3 index magnitude, the supplementation with polyunsaturated fatty acids also emerges as an important strategy for maintaining the optimum omega-3 index levels among professional athletes, exclusively in road cyclists, swimmers, wrestlers, and boxers falling into the high-risk group of the deficiency of omega-3 fatty acids.

Finally, even though this study has not found an association between the omega-6/omega-3 fatty acids ratio and the magnitude of the estimated omega-3 index, the existence of the evidence that the excessive consumption of refined oils (e.g., corn oil or soybean oil) and highly processed foods rich in omega-6 fatty acids may act as an inflammatory trigger that increases the requirements for the omega-3 fatty acids among athletes [133] can serve as a framework for further directions related to longitudinal and/or experimental studies aiming to confirm or decline the induction hypotheses mentioned above.

## Figures and Tables

**Table 1:** The sports-related characteristics according to the sport branches and sex of athletes.

Characteristics of Athletes	Anaerobic Sports		Aerobic Sports		*d* ^a^	*d* ^b^
	♂ (n = 68)	♀ (n = 8)	♂ (n = 85)	♀ (n = 39)		
Examination month	May–June		March–August		—	—
Age (in years)	19.8 [19.2; 20.4]	20.5 [16.6; 24.4]	19.8 [19.3; 20.3]	19.9 [19.1; 20.7]	0.01	0.2
Training experience (in years)	8.5 [7.6; 9.4]	8.2 [3.7; 12.6]	7.9 [7.0; 8.7]	7.3 [5.9; 8.6]	0.2	0.2
Exercise per week (in days)	5.9 [5.7; 6.1]	5.0 [3.6; 6.4]	5.9 [5.7; 6.0]	6.1 [5.9; 6.3]	0.1	−0.1
Single exercise sessions per day	1.7 [1.6; 1.9]	1.4 [0.8; 1.9]	1.6 [1.5; 1.7]	1.5 [1.4; 1.7]	0.2	−0.2
Duration of training (min per day)	184 [169; 199]	131 [86; 177]	185 [171; 200]	173 [155; 191]	−0.02	−0.2
Five training zones of intensity (%)						
1. AER strength endurance (PR: 120–140 bpm; LAK: <2 mmol/L)	12–18 ^1^		18–36 ^1^		—	—
2. AER strength (PR: 140–160 bpm; LAK: 2–4 mmol/L)	26–42 ^1^		37–42 ^1^		—	—
3. Cross AER + ANA glycolytic strength (PR: 160–180 bpm; LAK: 4–12 mmol/L)	32–49 ^1^		15–35 ^1^		—	—
4. ANA glycolytic strength (PR: ≥181 bpm; LAK: <21 mmol/L)	5–10 ^1^		3–10 ^1^		—	—
5. ATP−PC power (LAK: 1.5–6.0 mmol/L)	2–4 ^1^		1–3 ^1^		—	—

Data are presented as means ± 95% CIs [LB; UB] and as percentages ^1^; *d* ^a^—the effect size showing the standardized difference between the means of sports-related characteristics in anaerobic and aerobic male athletes; *d* ^b^—the effect size showing the standardized difference between means of sports-related characteristics in anaerobic and aerobic female athletes; AER—aerobic, ANA—anaerobic, ATP–PC—adenosine triphosphate–phosphocreatine, CI—confidence interval, bpm—beats per minute, LAK—lactate level, LB—lower bound, PR—pulse rate, UB—upper bound, ♂—males, ♀—females.

**Table 2:** Body composition of professional athletes.

Anthropometric Characteristics	Anaerobic Sports		Aerobic Sports		Norm		*d* ^a^	*d* ^b^
	♂ (n = 68)	♀ (n = 8)	♂ (n = 85)	♀ (n = 39)	**♂**	**♀**		
Height (cm)	188 [184; 192] *	170 [160; 180]	184 [182; 186]	168 [166; 170]	—	—	0.4	0.2
Body weight (kg)	80.4 [76.7; 84.1]	61.7 [52.5; 70.8]	76.6 [74.3; 78.9]	59.1 [56.6; 61.7]	—	—	0.2	0.2
Fatfree mass (kg)	66.4 [63.9; 68.9]	47.2 [41.6; 52.8]	63.2 [61.6; 64.8]	45.7 [44.0; 47.3]	—	—	0.4	0.3
Fat-free mass (% of BW)_BIA_	83.2 [82; 84.3] *	76.8 [73; 80.7]	82.7 [81.9; 83.4]	77.0 [75.9; 78.2]	75–85	70–80	0.1	−0.1
Body fat (kg)	14.1 [12.6; 15.6]	14.5 [10.1; 18.8]	13.5 [12.6; 14.4]	13.5 [12.3; 14.6]	—	—	0.1	0.2
Body fat (% of BW)_BIA_	16.8 [15.6; 17.9]	23.3 [19.5; 27.1]	17.3 [16.5; 18.0]	22.5 [21.3; 23.7]	10–14	20–24	−0.1	0.2

Data are presented as means ±95% CIs [LB; UB] and as percentages; *d* ^a^—the effect size showing the standardized difference between the means of anthropometric characteristics in anaerobic and aerobic male athletes; *d* ^b^—the effect size showing the standardized difference between the means of anthropometric characteristics in anaerobic and aerobic female athletes; BIA—the bioelectrical impedance analysis, BW—body weight, CI—confidence interval, LB—lower bound, UB—upper bound, ♂—males, ♀—females, *—*p*-value ≤ 0.05.

**Table 3:** Nutritional profile in athletes according to sports and the recommended daily allowances.

Nutrient Intake	Anaerobic Sports (n = 76)	Aerobic Sports (n = 124)	Requirements	*d* ^a^	*d* ^b^	*d* ^c^
Energy intake (kcal/day)	3900 [3602; 4199] *	3338 [3144; 3530]	3547	0.5	0.3	−0.2
Carbohydrates (g/kg of BW/day)	5.7 [5.3; 6.2]	5.3 [4.9; 5.7]	7–10 (8.5 ^1^)	0.2	−1.5	−1.6
Carbohydrates (% of EI)	45.6 [44.1; 47.1]	45.2 [43.7; 46.7]	45–65 (55 ^1^)	0.1	−1.4	−1.2
Protein (g/kg of BW/day)	1.8 [1.6; 1.9]	1.7 [1.6; 1.8]	1.4–2.0 (1.7 ^1^)	0.1	0.3	0.2
Protein (% of EI)	14.1 [13.5; 14.7]	14.5 [13.9; 15.1]	12–20 (16 ^1^)	−0.1	−0.8	−0.5
Fat (g/kg of BW/day)	2.2 [2.1; 2.4]	2.1 [1.9; 2.3]	—	0.2	—	—
Fat (% of EI)	40.3 [38.8; 41.8]	40.4 [38.9; 41.7]	25–35 (30 ^1^)	0.01	1.6	1.3
SFA (g/day)	59.7 [55.4; 64.0] *	51.3 [47.2; 55.4]	—	0.4	—	—
SFA (% of EI)	14.1 [13.5; 14.7]	13.7 [13.1; 14.4]	≤10	0.1	1.6	1.0
PUFA (g/day)	27.2 [24.6; 29.8]	23.8 [21.3; 26.3]	—	0.2	—	—
PUFA (% of EI)	6.2 [5.9; 6.6]	6.2 [5.8; 6.7]	6–10 (8 ^1^)	0.01	−1.2	−0.7
ω-3 FA (g/day)	1.4 [1.3; 1.5]	1.3 [1.1; 1.4]	—	0.2	—	—
ω-3 FA (% of EI)	0.3 [0.3; 0.4]	0.3 [0.3; 0.4]	≥2	0.02	−12	−6.0
ω-6 FA (g/day)	25.2 [22.7; 27.8] *	21.8 [19.4; 24.2]	—	0.3	—	—
ω-6 FA (% of EI)	5.8 [5.4; 6.1]	5.7 [5.2; 6.1]	5–8 (6.5 ^1^)	0.04	−0.5	−0.3
ω-6/ω-3 FA ratio	18.5 [16.9; 20.1]	18.3 [16.9; 19.7]	4	0.02	2.1	1.8

Data are presented as means ±95% CIs [LB; UB]; *d* ^a^—the effect size showing the standardized difference between the means of macronutrient intakes in anaerobic and aerobic athletes; *d* ^b^—the effect size showing the standardized difference between the means of the quantities of macronutrients consumed by anaerobic athletes and the recommended daily allowances; *d* ^c^—the effect size showing the standardized difference between the means of the quantities of macronutrients consumed by aerobic athletes and the recommended daily allowances; EI—energy intake; FA—fatty acids; PUFA—polyunsaturated fatty acids, SFA—saturated fatty acids; ^1^—measure of central tendency; *—*p*-value ≤ 0.05.

**Table 4:** Association between the nutritional intake, dietary habits and supplementation and the estimated ω-3 index (%) as a dependent variable (multiple regression analyses).

Independent Variables	β (SE)	95% CI [LB; UB]	VIF	*p*
ω-3 FA (% of EI)	0.8 (0.4)	[0.1; 1.6]	4.6	0.049
ω-6 FA (% of EI)	0.01 (0.04)	[−0.1; 0.1]	3.4	0.768
ω-6/ω-3 FA ratio	−0.01 (0.01)	[−0.03; 0.02]	4.4	0.576
Duration of PUFA supplementation	1.5 (0.1)	[1.3; 1.6]	1.0	<0.001
Frequency of fish products consumption	1.1 (0.1)	[1.0; 1.2]	1.0	<0.001

Model: F_7.192_ = 105.7, *p* < 0.001, *R*^2^ = 0.79. The linear regression model was adjusted to the sports branches and sex of the athletes; CI—confidence interval, EI—energy intake, F—the F-statistic, FA—fatty acids, LB—lower bound, PUFA—polyunsaturated fatty acids, *R*^2^—the *R*-Squared (*R*^2^ is appropriate: ≥0.2 (20%)), UB—upper bound, VIF—the variance inflation factor (the VIFs are appropriate if fluctuate from 1 to 5), *p*—*p*-value.

## Data Availability

The data presented in this study are available on request from the corresponding author unless sharing them would go against ethical considerations, such as study participants’ consent and privacy.

## Abbreviations

The following abbreviations are used in this manuscript:

AER	Aerobic
ANA	Anaerobic
ARA	Arachidonic acid
ATP–PC	Adenosine triphosphate–phosphocreatine
BIA	Bioelectrical impedance analysis
Bpm	Beats per minute
BW	Body weight
CI	Confidence interval
D	Effect size
DHA	Docosahexaenoic acid
EI	Energy intake
EPA	Eicosapentaenoic acid
FA	Fatty acids
LAK	Lactate level
LB	Lower bound
LTOK	National Olympic Committee
M	Mean
PR	Pulse rate
PUFA	Polyunsaturated fatty acids
RBC	Red blood cells
SD	Standard deviation
SE	Standard error
SFA	Saturated fatty acids
STROBE	Strengthening the reporting of observational studies in epidemiology
UB	Upper bound
VIF	Variance inflation factor
WHO	World Health Organization
ω-3I	Omega-3 index
